# Predicting gene ontology from a global meta-analysis of 1-color microarray experiments

**DOI:** 10.1186/1471-2105-12-S10-S14

**Published:** 2011-10-18

**Authors:** Mikhail G Dozmorov, Cory B Giles, Jonathan D Wren

**Affiliations:** 1 Arthritis and Clinical Immunology Research Program, Oklahoma Medical Research Foundation; 825 N.E. 13th Street, Oklahoma City, Oklahoma 73104-5005, USA

## Abstract

**Abstract:**

**Background**

Global meta-analysis (GMA) of microarray data to identify genes with highly similar co-expression profiles is emerging as an accurate method to predict gene function and phenotype, even in the absence of published data on the gene(s) being analyzed. With a third of human genes still uncharacterized, this approach is a promising way to direct experiments and rapidly understand the biological roles of genes. To predict function for genes of interest, GMA relies on a guilt-by-association approach to identify sets of genes with known functions that are consistently co-expressed with it across different experimental conditions, suggesting coordinated regulation for a specific biological purpose. Our goal here is to define how sample, dataset size and ranking parameters affect prediction performance.

**Results**

13,000 human 1-color microarrays were downloaded from GEO for GMA analysis. Prediction performance was benchmarked by calculating the distance within the Gene Ontology (GO) tree between predicted function and annotated function for sets of 100 randomly selected genes. We find the number of new predicted functions rises as more datasets are added, but begins to saturate at a sample size of approximately 2,000 experiments. For the gene set used to predict function, we find precision to be higher with smaller set sizes, yet with correspondingly poor recall and, as set size is increased, recall and F-measure also tend to increase but at the cost of precision.

**Conclusions**

Of the 20,813 genes expressed in 50 or more experiments, at least one predicted GO category was found for 72.5% of them. Of the 5,720 genes without GO annotation, 4,189 had at least one predicted ontology using top 40 co-expressed genes for prediction analysis. For the remaining 1,531 genes without GO predictions or annotations, ~17% (257 genes) had sufficient co-expression data yet no statistically significantly overrepresented ontologies, suggesting their regulation may be more complex.

## Background

As the availability and abundance of microarray data has grown across the major microarray data repositories, such as Gene Expression Omnibus (GEO) [1], ArrayExpress [2], and the Stanford Microarray Database [3] to encompass hundreds of thousands of experiments now, there is an increased interest in methods of mining this data. One approach has been to conduct what might be termed a global meta-analysis, which differs from traditional meta-analysis of experimental datasets that are normally undertaken to increase sample size by using highly similar experimental conditions and tissue types. The motivation behind a meta-analysis of heterogeneous data is to focus on gene-gene transcriptional patterns rather than experiment-experiment patterns.

In any given microarray experiment, there are likely many different processes going on at once when comparing experiment versus control. For example, even when one condition, such as oxidative stress, is induced, cells experience changes in multiple processes such as enzymatic activity, chromatin structure, apoptosis-related signaling, antioxidant production, etc. Identifying gene pairs that are consistently differentially expressed with each other across many different conditions permits an analysis of which of the many genes involved in these different processes are specific to each other outside of individual experimental conditions (e.g., apoptosis can be induced in response to many different stimuli).

### Using patterns of consistent co-expression to predict function

The generation of high-throughput data provides the opportunity to examine patterns across datasets to identify correlations [4]. Analysis of co-expression networks to try to identify regulatory patterns and modularity in co-expression dates back to early studies in yeast [5,6] and soon thereafter in higher organisms [7]. One of the motivations in identifying these patterns is that they can be used to predict gene function [8,9], as well as potential roles for non-coding elements [10]. Different methods of identifying gene-gene correlations are used, of which Pearson’s has been widely used[11-14], but also other patterns such as dividing patterns into parallel/anti-parallel [15] and Boolean quadrants [16] have been useful. It’s becoming clear that methods to study gene-gene co-expression patterns across unrelated experiments can tell us about the underlying genetic regulation, which has broad implications. For example, by establishing what could be considered “normal” gene-gene regulation one can than try to detect abnormal or disease-related disturbances [17]. In particular, with approximately 34% of human genes still without known function [15,18], it is important to develop methods to accurately predict function. The situation in human is not unique – the fraction of still uncharacterized genes ranges from ~38% in mouse [19] to ~17% in perhaps the best-studied eukaryotic organism of all, yeast [20].

**Figure 1:**
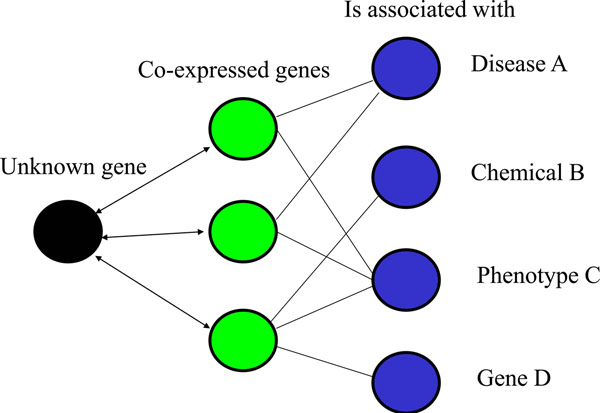
**“Guilt by association” approach to inferring gene function**. Co-expressed genes can be interrogated for commonalities to identify probable associations.

The general approach to inferring associations using guilt by association is outlined in Figure 1. Here, associations can be inferred by analyzing a set of genes that are consistently co-expressed with a query gene across heterogeneous conditions. These genes can then be analyzed for what they have in common. Using GO, this would yield predicted associations with molecular function, biological process and cellular components. Literature-based analysis software such as IRIDESCENT [21-23] could also be used to identify phenotypes, diseases and other entities such as drugs and/or chemicals that are also associated with the genes in the literature. Studies have shown that this approach can predict GO categories [24], but at this point, it’s not known how accurate this type of approach is at inferring different associations (e.g., disease, phenotype, cellular location, etc).

Our initial study used 3,600 human 2-color arrays, which yielded a 34% direct hit rate (i.e., the predicted GO category was the same as the known GO category) [15]. On the surface, this may appear to be a low accuracy, but because GO annotation lags the literature and our knowledge of genes is biased towards a select few [15,18], it is difficult to estimate the false-positive rate in this type of analysis. That is, it is not clear how many predicted functions are simply not known. For example, we have experimentally examined some of the predicted functions made from this 2-color analysis and have found them to be generally accurate (manuscripts in preparation) and have published the results of one of these studies [25]. In this initial study, gene expression was categorized into genes that were expressed in parallel (i.e., tended to be up-regulated and down-regulated together), anti-parallel (i.e., when one was up-regulated, the other tended to be down-regulated and vice versa) and not differentially expressed. Here, we first want to establish whether or not similar parameters can be used for 1-color arrays which do not display differential expression like their 2-color counterparts, but merely expression or non-expression, an important technical distinction [26]. Second, we want to know how the set size of genes used for inference affects precision and recall (e.g., in Figure 1, 3 genes are shown as being used to infer function). Finally, we want to know what the relative contribution of the ranked genes is to the process of inference – that is, do the top genes contribute to more accurate predicted functions.

## Methods

### Obtaining experimental and annotation data

From NCBI’s Gene Expression Omnibus (GEO) database [27], GEO Dataset (GDS) files were obtained. Analysis was restricted to datasets that came that contained the following headers: dataset_sample_organism=“homo sapiens”, dataset_type=”nucleotide” or “gene expression”, dataset_channel_count=”single” or “1”, and dataset_value_type=”count”. This ensures only raw data from one-channel human microarray samples were processed. All dataset annotation, gene annotation, GO annotation data were stored in an MS Access database and queued from custom programs written in Visual Basic 2010.

Gene Ontology annotations were downloaded on 3/14/2011 from NCBI [28]. Only human (Taxonomy ID 9606) gene to GO mapping was used in the current study. An enrichment of GOs within a set of co-expressed genes was calculated using chi-square test. The Gene Ontology tree file in Open Biomedical Ontology (OBO) format v1.2 was downloaded from Gene Ontology [29] on 3/14/2011.

### Normalization & preprocessing of microarray experimental data

Datasets with mean or median values of 0 were excluded, as well as those with mean to median ratio <= 1. Data for each experiment were sorted and distribution of expression values around maximum was examined. No more than 0.1% of maximum expression showed abnormally high expression values, due to either technical of software errors. These values were selected, a minimum (flooring) value among then wad identified, and all these values were set to this flooring value. As such, abnormally high expression values were brought to a reasonably high expression level. Each experimental dataset was then adjusted to fit within 0 to 10,000 range to make data ranges comparable across datasets. Distributions of the data fit within 0-10,000 range were quantile-normalized [30], which makes them equal and amenable for defining of the noise threshold used in the subsequent analysis. A total of 2,325 GDS files were downloaded, out of which 587 contained raw gene expression data from human single-channel microarrays, while others contained data for other organisms or from two-color microarrays.

Probe mapping was done by mapping gene names and accession numbers downloaded from NCBI [31] to unique Entrez ID identifiers. Within each experiment for multiple probes mapping to the same gene, the maximum expression value was used under the assumption that they may reflect different exons and the most intense signal is likely to come from constitutive exon. Probe identifiers were mapped to Entrez IDs, totaling 20,813 genes. All data were assembled in a matrix with columns representing EntrezIDs and rows containing expression values for a given experiment.

### Metrics used to rank gene-gene co-expression patterns

Gene expression statistics for each gene-gene pair were calculated from across all 13,000 experiments, but only when both genes were present in the same microarray experiment. Several parameters were measured as potential means of ranking co-expression specificity and consistency. “Purity” refers to the general behavioral tendency of two genes to either be expressed above noise level defined after quantile normalization step, or not expressed with each other (e.g., 100% Purity means the two genes are always expressed or not expressed together), regardless of magnitude of expression. “Total” is the total number of times the two genes (A and B) were expressed. Mutual information measure (MIM) is calculated as log2(P(A|B)/(P(A)*P(B)), where P(A) and P(B) are the probabilities of gene A and B being expressed, respectively, and P(A|B) is the conditional probability of gene A being expressed when gene B is expressed. R2 is Pearson’s correlation coefficient squared, calculated using Alglib package [32]. The advantages and drawbacks of each measure are shown in Table 1. Correlation score (equation 1) was calculated for each pair and selected number of genes with highest score and having at least one GO annotation was used for prediction analysis. As a control, a set of randomly selected genes was used for predictions.

**Table 1:** Parameters used for prediction analysis and their properties

Parameter	Information it gives	Drawback
Total = Frequency of gene pair co-expression	Total number of times a gene pair is expressed, excluding missing values	Some genes are expressed more frequently than others

MIM = Mutual Information Measure	Specificity of co-expression	When Total is small, MIM can be artificially high

R^2^ = Pearson’s Correlation Coefficient	Correlation between gene pair expression levels	Will detect global, but not conditional, co-regulation. Also, non-expression is far more common than expression, biasing R^2^ (e.g., 2 genes never expressed will show perfect correlation)

P = Purity	When co-expression “behavior” is described in terms of discrete categories, purity reflects a relative breakdown of behavioral observations	Information can be lost when discretizing a continuous variable

*Equation 1*: 	SCORE = log_2_MIMpara * Purity^2^ * Total * R^2^

### Gene Ontology concordance and divergence analysis

To test how the number of microarray experiments and the number of co-expressed genes used for predictions influence prediction power for a set of randomly selected 100 genes having from 1 to 20 officially annotated ontologies. To determine how well the “guilt by association” is working for a given query gene, we first rank a set of co-expressed genes that we believe should be most representative of the function of the query gene. Then, these genes are analyzed for what GO categories they have in common by GO enrichment analysis. The enriched categories are the predicted GO categories, which are then compared to the known (annotated) GO categories to see how far they are on the GO tree from the known categories. The minimum distance to the nearest GO category is used in cases where more than one GO category exists for a gene (which is common). True positive hits (TP) were defined as the number of predicted GO categories correctly inferred for a query gene (defined as a distance of zero between the predicted and known GO category). False positives (FP) were predicted GO categories with a distance > 0. False negatives (FN) were defined as the total number of GO categories assigned to a target gene that were not predicted.

Recall, precision, and F-measure were calculated as follows:

*Equation 2*: 	Recall = TP/(TP+FN)

*Equation 3*: 	Precision = TP/(TP+FP)

*Equation 4*: 	F-measure = 2*Precision*Recall/(Precision+Recall)

To explore how predicted ontologies for a given gene within an acyclic GO graph correspond to known ontologies we calculated the shortest distance between predicted and annotated ontologies for each gene, as was done in our previous work [15]. Traversing the GO tree to identify the shortest path between GO categories using “is_a” relationships was conducted using Dijkstra’s algorithm, which is part of the QuickGraph package v3.0 (http://quickgraph.codeplex.com/). Venn diagram was build using Venny tool [33].

## Results

### Examining scoring metrics to rank co-expressed genes from 1-color data

Out of 20,813 genes, 5,720 (27.5%) did not have GO annotations. We examined several different ways of predicting functions of these unannotated genes from annotated gene-gene co-expression sets. There are several different parameters that can be used for selection of co-expression sets, either individually or in combination (Table 1).

**Figure 2:**
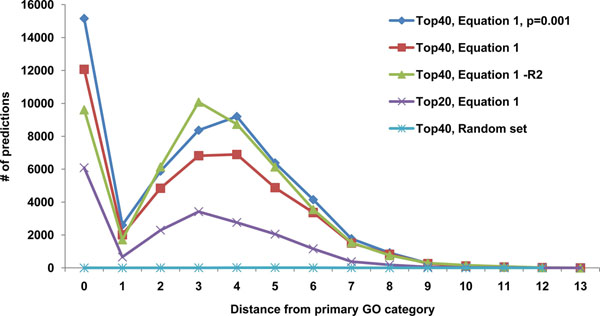
**Examining different metrics to predict GO categories for a gene using a set of its co-expressed genes.** Addition of Pearson’s correlation coefficient to calculation of co-expression score (equation 1, see also [5]) increased the number of predicted ontologies equal to canonical GOs previously assigned to a gene (number of direct hits, (red vs. light blue lines). Using top 20 co-expressed genes instead of top 40 decreased the number of direct hits (red vs. dark blue lines).

A GO concordance and divergence analysis was run to see how well different ranking metrics performed to predict function (see Methods). Several permutations were tested, the results are shown in Figure 2. Using the Top 40 co-expressed genes instead of top 20 nearly doubled the number of direct hits (number of predicted ontologies that correspond to annotated ontology categories). However, the number of indirect hits (predicted ontologies that are statistically significant, yet are at a distance > 0 from any known ontology category) increased proportionally. Omitting Pearson’s correlation coefficient led to decrease in the number of direct hits yet, interestingly, increased the number of statistically significant indirect hits. Surprisingly, using a less strict p-value cut-off (p < 0.001 rather than p < 0.0001) for enrichment analysis led to an increase in number of both direct and indirect hits.

Besides calculating overall F-measure we investigated recall and precision in each of the three separate GO namespaces. Using top 40 co-expressed genes predictions for GO annotations in “cellular component” namespace performed best (F-measure=0.16), followed by predictions in “molecular function” namespace (F-measure=0.11). Although the “biological process” namespace had the largest number of predicted GO annotations, general performance was not as high (F-measure=0.07).

### A relatively small subset of co-expressed genes have the most predictive power

**Figure 3:**
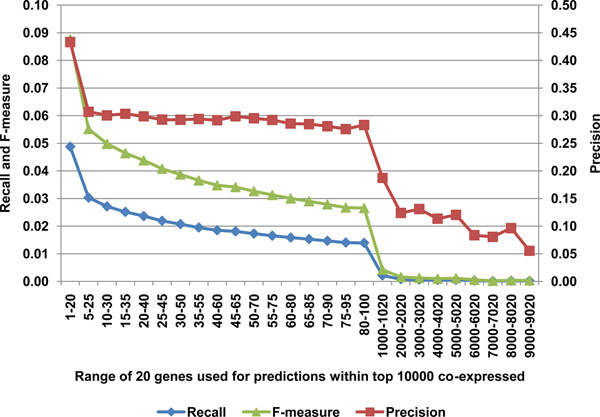
**Influence of top co-expressed gene selection on prediction power.** Only top co-expressed genes achieved best balance of recall and precision (F-measure), which dropped dramatically as less well co-expressed genes were selected.

Previous studies used different numbers of genes for the guilt-by-association analysis, but have not determined the relative contribution of genes towards predictive power. Doing so will tell us whether effort is better spent identifying a small, informative subset (or module) that will have the most predictive power, suggesting that expanding further than this small subset would have minimal effects on precision and recall. Alternatively, if performance drops linearly, this would argue against modularity and suggest that genes are more intertwined in general. We examined how the selection of gene subsets from among the top ranked genes affected functional predictions. We ranked the top 100 co-expressed genes selected using equation 1 and used subsets of 20 genes, gradually sliding down from the top ones in steps of 5. Figure 3 shows that performance declines rapidly once the selection window moves out of the top 5 genes. As expected, precision and recall deteriorate while moving down the list of co-expressed genes, eventually reaching the performance levels of randomly selected gene lists (Recall_Random_=0.0013, Precision_Random_=0.0854) around the ~2000th -2020th co-expressed gene mark. Because performance declines rapidly after the first selection window, these results are consistent with the idea that gene expression is modular, and suggest that identification of module boundaries could improve predictive performance.

### Effects of set size on recall and precision

**Figure 4:**
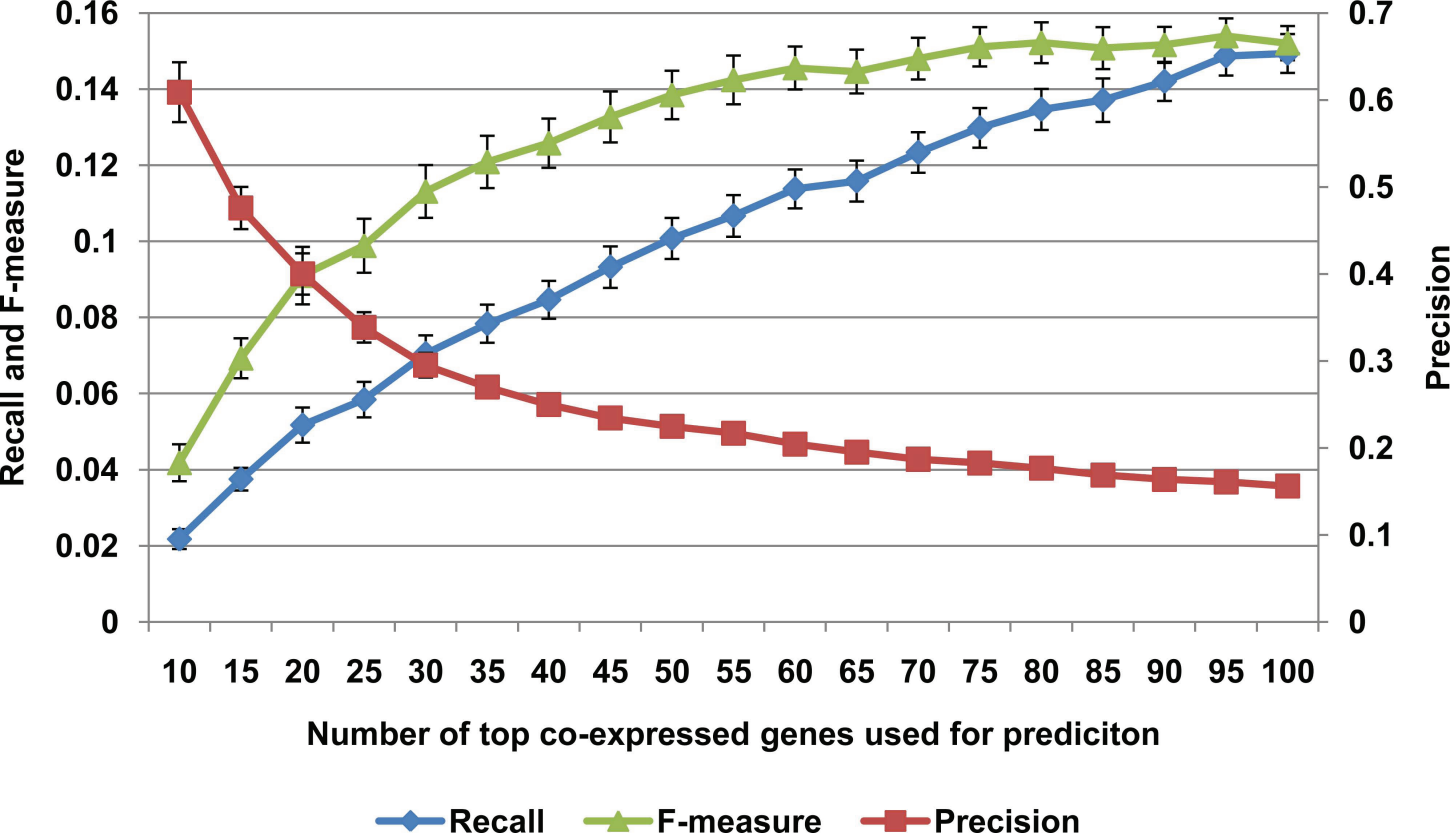
**Size of co-expressed gene sets on sensitivity and recall (n=20).** Selection of top 40 co-expressed genes yields best tradeoff between sensitivity and precision.

We then analyzed the precision and recall associated with set size to identify an optimal number of genes to select for inferring gene function (see Figure 1). First, 100 genes with between 1 and 20 annotated GO categories were randomly chosen for analysis. Then, for each gene randomly chosen, genes were ranked using equation 1 and a set of co-expressed genes of varying sizes was submitted for GO enrichment analysis (i.e., the top n genes where n is shown on the x-axis in Figure 4). We found that the sensitivity for predicting GO categories increased as larger sets of co-expressed genes were used, but at the expense of precision. The F-measure does not appear to reach a maximum until approximately 90 genes, but around 30 genes, the rate of return begins to decrease fairly rapidly.

**Figure 5:**
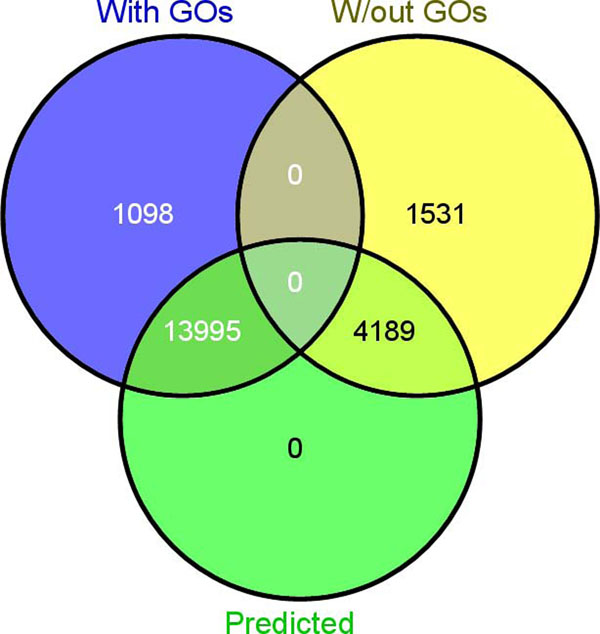
Venn diagram of overlap between the number of genes with predicted ontologies (“Predicted”) with annotated (“With GOs”) and unannotated (“W/out GOs”) genes

Using the top 40 co-expressed genes, predicted GO annotations were obtained for 4,189 out of 5,720 (73%) genes. The rest of them, 1,531 genes, were expressed above noise in less than 50 experiments and/or their co-expressed genes were not enriched in any ontologies (Figure 5).

### Effect of sample size on predictive power

**Figure 6:**
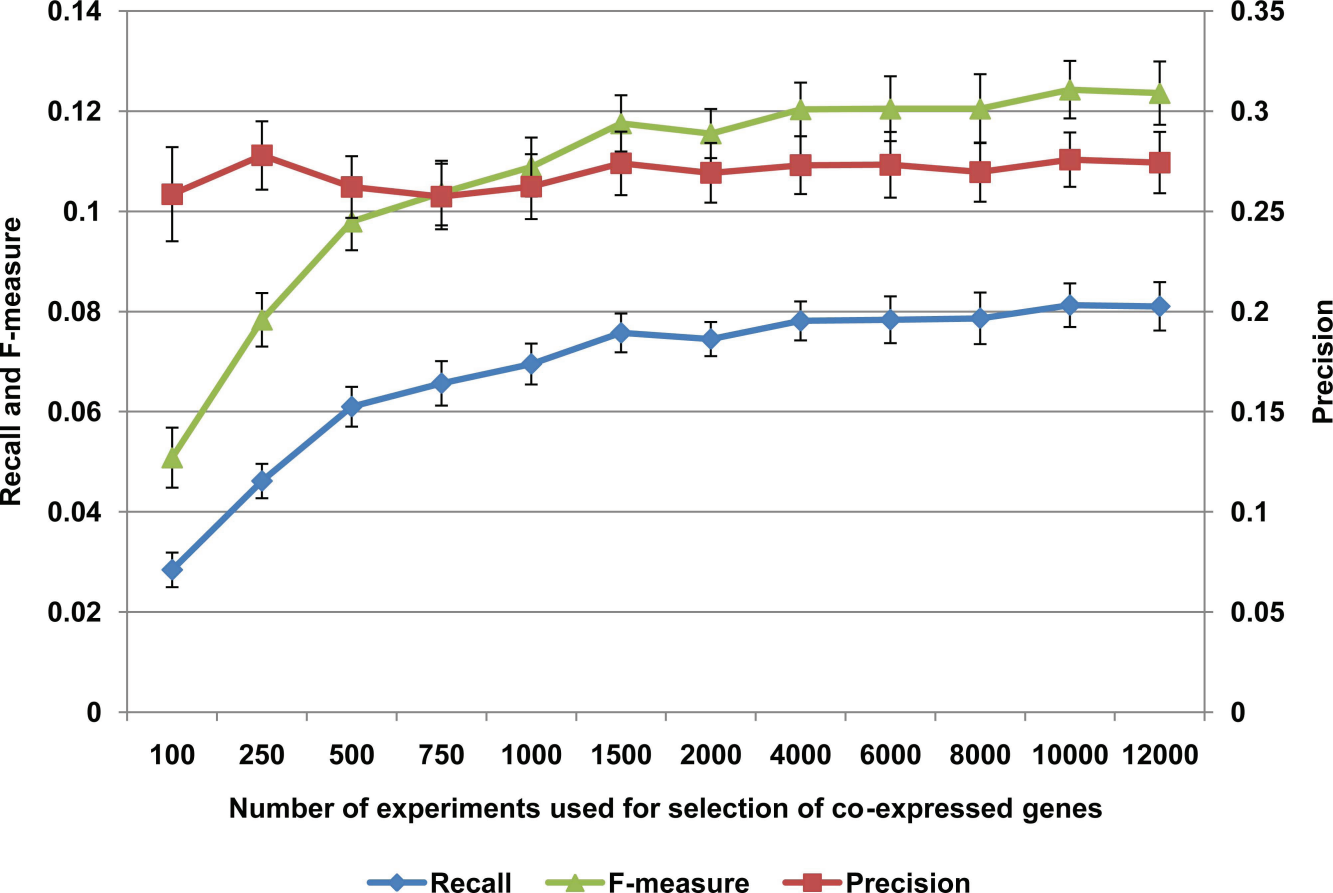
**In functional prediction performance per microarray experiment analyzed (n=20).** Recall and precision of prediction, encapsulated in F-measure, show signs of saturation around 2,000 experiments.

To examine how GMA performance was affected by the number of experiments processed, we randomly selected 100 genes for analysis and then processed a variable number of randomly selected experiments from within the 13,000 experiments analyzed using top 40 co-expressed genes for prediction. We hypothesized that performance should increase with sample size, but eventually peak as observed co-expression patterns begin to recur. We found that this is indeed the case, with an F-measure showing signs of saturation around 2,000 experiments (Figure 6).

## Discussion

Two-color microarrays contain information about co-repression of gene pairs, but in 1-color arrays the measurement focuses on expression rather than differential expression and information is only obtained on co-induction of gene pairs. Non-expressed gene pairs in 1-color arrays are normally uninformative since most genes are not expressed at any given point in time. The ability to use a large set of heterogeneous microarray datasets as a means of studying gene-gene co-expression and predicting function has been demonstrated by us [15,25] and others [6,7,24] previously, but much of the details underlying why and how the approach works has not been explored.

Our metrics for selection of co-expressed genes (Equation 1) have been validated in the previous work [15], however, for one-color microarray data addition of Pearson’s correlation coefficient increased the performance of predictions. The main limitation of our work is incomplete GO annotations, which led to relatively low F-measure. Also, other metrics for finding similarities among GO annotations [34] can be used to improve statistical calculations. We expect the quality of predictions to increase as new GO annotations will be added.

We observed the F-measure of predictions begin to taper off around 2,000 experiments analyzed (Figure 6). While rarely expressed transcripts will likely benefit from more experiments analyzed, this finding is important because it tells us that, for most genes, there is more than enough experimental data to make predictions. It is also important because, as more data is accumulated on non-coding RNA (ncRNA) expression, it suggests approximately how many experiments we will need to begin correlating ncRNA expression with gene function. Currently, among the GSE files deposited in GEO there were 370 annotated as “non-coding RNA profiling by array” at the time of this writing. Interestingly, precision remained constant independently of the number of experiments used (Figure 6), suggesting overall that only a small amount of co-expression data is sufficient to garner accurate predictions for genes that are included within these experiments, but that more data is required to produce predictions for transcripts that are more rarely expressed or are included within few platforms, resulting in a more gradual increase in recall.

Another important observation is that precision drops quickly once genes outside the top ranked group are chosen. Although not unexpected, it is useful to know that most of the informative gene pairs are within a relatively small group or module. This suggests that it is important to algorithmically identify and characterize these modules to maximize our ability to infer associations from these co-expressed gene sets.

Interestingly, we observed an overall trend that genes tend to co-express with other genes from the same subfamilies. For example, among top 20 genes best co-expressed with LILRB1 (leukocyte immunoglobulin-like receptor, subfamily B member 1) there were LILRB3, LILRA6, LILRA3 and others alike, such as PILRA (paired immunoglobulin-like type 2 receptor alpha). This not only further strengthens “guilt by association” principle but also explains why some poorly annotated genes did not have any predictions. A poorly annotated gene tends to be co-expressed with other poorly annotated genes. As such, the top co-expressed genes without ontologies would be discarded (because they cannot be used to infer GO category), while annotated genes further down the list already don’t have enough precision for functional prediction (see Figure 3).

Precision and recall differed for different GO namespaces. Seemingly counter intuitively, “cellular component” has the highest precision/recall rates, and “biological process” has the lowest. An explanation lies in the total number of annotations in a given category. “Cellular component” has the lowest number of annotations and subcategories, is easier to establish experimentally than either molecular function or biological process, and thus the rate of true positives would be higher. “Biological process” on the other hand has largest number of annotations and, other parameters equal, the rate of direct hits in relation to the total number of annotations would be lower. “Molecular function” had intermediate number of annotations and intermediate precision/recall rates.

## Conclusions	

A global analysis of gene-gene co-expression behavior is a promising means of predicting gene function, particularly for the third of human genes that are still uncharacterized and for those that are only sparsely characterized. Our examination of how much data is needed to effectively conduct these analyses and how different parameters affect the precision and recall of inference will help enable this approach.

## Competing interests

The authors declare that they have no competing interests.

## Authors' contributions

JDW conceived of the project. MGD designed, implemented and tested Gene Ontology concordance and divergence analysis for one-color microarray data. CGB implemented Gene Ontology acyclic graph traversing. All authors wrote the manuscript. All authors read and approved the final manuscript.
